# Malaria in Greece: From Ancient Scourge to Eradication

**DOI:** 10.7759/cureus.103294

**Published:** 2026-02-09

**Authors:** Magdalini K Christodoulou

**Affiliations:** 1 School of Health Sciences, University of Thessaly, Larissa, GRC

**Keywords:** greece, malaria elimination, plasmodium vivax, public health, re-emergence

## Abstract

Malaria has played a profound role in the historical, social, and public health development of Greece, from antiquity to the modern era. This narrative review examines the long-term trajectory of malaria in Greece, tracing its presence from early medical descriptions through periods of endemicity, the national eradication campaign, and the subsequent re-emergence of locally acquired cases in the early 21st century. The review highlights the multifaceted strategies that led to successful elimination, including vector control, antimalarial treatment, environmental interventions, and sustained surveillance systems. It also explores the factors that contributed to the temporary re-establishment of transmission following eradication, emphasizing the ongoing vulnerability of malaria-free regions in the context of global mobility and persistent competent vectors. Greece’s experience demonstrates that malaria elimination is achievable through coordinated, long-term public health efforts, but also underscores the necessity of continuous surveillance and rapid response capacity to prevent re-establishment of transmission. The lessons drawn from this historical and epidemiological experience remain highly relevant for contemporary malaria elimination and post-elimination strategies worldwide.

## Introduction and background

Malaria has profoundly shaped human civilization, influencing military campaigns, demographic patterns, and economic development across continents [1]. Despite remarkable advances, malaria remains a formidable disease. In Greece, malaria’s documented presence extends to antiquity, making it among the oldest recorded diseases in European medical literature. Hippocrates provided systematic clinical descriptions in the fifth century BCE, recognizing distinct fever patterns corresponding to different *Plasmodium* species millennia before scientists understood the disease’s etiology [2]. During the early 20th century, Greece had the highest malaria prevalence in Europe [3]. The 1905 epidemic affected approximately 960,000 people, nearly one-fifth of the population [4]. The arrival of 1.2 million refugees following the 1922 Greco-Turkish War catastrophically exacerbated the situation, with malaria accounting for approximately 70% of refugee deaths in northern rural areas during 1923 [5,6]. Yet Greece’s malaria story ultimately chronicles triumph. Through the dedicated efforts of Greek physicians, innovative institutions like the Greek Anti-Malaria League (1905), strategic collaboration with international organizations, including the Rockefeller Foundation and the League of Nations, and comprehensive post-World War II eradication programs, Greece achieved complete elimination by 1974 [7]. This review examines malaria’s historical trajectory in Greece and explores lessons relevant to contemporary global health efforts.

Methodology

This narrative review employed a comprehensive literature search strategy to examine the historical and epidemiological trajectory of malaria in Greece from antiquity through eradication to contemporary re-emergence. We searched PubMed, Scopus, Google Scholar, and the World Health Organization (WHO) databases using the following search terms in various combinations with Boolean operators (AND, OR): “malaria,” “Greece,” “eradication,” “elimination,” “Plasmodium vivax,” “historical,” “epidemic,” “1905,” “refugee crisis,” “1922,” “DDT,” “public health,” “re-emergence,” “2011,” “2012,” and “surveillance.” The search covered publications from database inception through December 2024, with no language restrictions.

Inclusion criteria encompassed (i) peer-reviewed articles, books, and official WHO reports addressing malaria in Greece, (ii) historical documents and epidemiological studies describing malaria prevalence, control efforts, or eradication campaigns, (iii) studies examining the 2011-2012 malaria re-emergence, and (iv) systematic reviews and meta-analyses relevant to malaria elimination strategies. Exclusion criteria included (i) studies focusing exclusively on malaria outside Greece without a comparative context, (ii) conference abstracts without full-text availability, and (iii) duplicate publications. The initial search yielded 187 potentially relevant publications. After removing duplicates and screening titles and abstracts, 78 articles underwent full-text review. Following detailed assessment against inclusion criteria, 25 sources were selected for inclusion in this review, comprising peer-reviewed journal articles (n=19), historical monographs (n=3), and WHO official reports (n=3). Data extraction focused on historical context, epidemiological patterns, intervention strategies, eradication campaign components, post-eradication surveillance, and the 2011-2012 outbreak response.

## Review

Historical background

The earliest systematic descriptions of malaria appear in the Hippocratic Corpus from the fifth and fourth centuries BCE [2]. Hippocrates classified fevers by periodicity, recognizing patterns corresponding to different *Plasmodium* species: tertian fevers (48-hour cycles from *Plasmodium vivax*), quartan fevers (72-hour cycles from *Plasmodium malariae*), and severe irregular manifestations consistent with *Plasmodium falciparum* cerebral malaria [8]. The Hippocratic texts documented the disease’s seasonal nature and introduced the concept of “miasma” (poisonous vapors) that cause disease. While incorrect regarding transmission, this theory correctly identified environmental associations with marshlands where Anopheles mosquitoes breed.

During Roman times, malaria devastated Mediterranean populations, particularly in the Roman Campagna. Some historians argue that endemic malaria contributed to the Western Roman Empire’s decline by weakening populations and reducing military effectiveness [9]. Throughout the Byzantine and Ottoman periods, malaria remained endemic across Greece, fundamentally shaping settlement patterns and economic activities.

Following Greek independence (1821-1829), malaria remained endemic across lowland regions. The disease severely reduced agricultural productivity, creating self-reinforcing poverty traps where disease caused poverty while poverty prevented control investments. High childhood mortality and pregnancy complications contributed to demographic stagnation.

Modern scientific understanding began when Laveran observed the malaria parasite in 1880 [10], definitively establishing the disease’s parasitic nature. Ronald Ross demonstrated mosquito transmission in 1897-1898 [11], identifying the mosquito as a targetable link in the transmission chain. These discoveries revolutionized malaria control thinking.

The eradication campaign

Early Control Efforts (1905-1945)

The catastrophic 1905 epidemic catalyzed an organized response. Greece established the Greek Anti-Malaria League in 1905 and a quinine monopoly in 1908, ensuring affordable access to treatment [4]. The 1922 refugee crisis brought unprecedented challenges. International assistance became crucial-the Rockefeller Foundation launched major initiatives during the 1920s-1930s, providing technical expertise and training [12], while the League of Nations supported control programs [13].

Comprehensive Eradication Strategy (1946-1974)

Post World War II, Greece embraced the WHO’s global malaria eradication strategy, launched in the mid-1950s [14]. The program received substantial international assistance through Marshall Plan funding. Greece’s success resulted from an integrated strategy addressing multiple transmission aspects simultaneously:

Indoor residual spraying with dichlorodiphenyltrichloroethane (DDT) dramatically reduced mosquito populations and shortened lifespan below the extrinsic incubation period. While later controversial environmentally, targeted indoor spraying used smaller quantities than agricultural applications and proved instrumental in achieving elimination. Mass treatment involved systematic case detection and treatment with chloroquine and primaquine for radical cure, particularly crucial for *P. vivax* dormant liver stages [15]. Treating the human reservoir prevented ongoing transmission. Environmental engineering permanently drained marshes and eliminated breeding sites through land reclamation. These modifications complemented insecticide spraying and reduced long-term transmission risk. Surveillance systems continuously monitored progress, identified remaining foci, detected imported cases, and enabled rapid response.

Critical success factors included sustained political commitment ensuring program continuity, adequate funding supplemented by international assistance, well-trained personnel, comprehensive surveillance enabling adaptive management, and operational flexibility. After nearly three decades of sustained effort, WHO officially certified Greece as malaria-free in 1974 following a rigorous epidemiological review [16]. In 1975, the WHO declared the entire European region malaria-free for the first time in recorded history.

Post-eradication challenges

Imported Cases and Surveillance (1975-2010)

Following eradication, Greece detected exclusively imported cases among travelers and migrants. Between 1975 and 2010, Greece documented 1,419 laboratory-confirmed imported cases, with annual numbers fluctuating between 19 and 76 [17]. Analysis revealed *P. falciparum* in 44% and *P. vivax* in 37% of cases. Imported cases predominantly affected males (79%) and individuals aged 18-40 years (72%).

The 2011-2012 Re-Emergence

Despite decades of malaria-free status, Greece experienced an unexpected outbreak of locally acquired *P. vivax* malaria in 2011-2012. During summer 2011, authorities identified 27 locally acquired cases among Greek citizens with no travel history in the Evrotas agricultural area of Laconia, Peloponnese [18]. Investigation revealed indigenous *Anopheles sacharovi* mosquitoes had become infected by feeding on immigrant agricultural workers from endemic countries harboring *P. vivax* infections [19]. Between 2009 and 2012, approximately 84 locally acquired cases were documented, representing the first sustained local transmission since eradication [20].

The public health response was swift and comprehensive: dramatically intensified surveillance with active case detection, prompt diagnosis, radical treatment with chloroquine plus primaquine, targeted vector control including indoor residual spraying, and mass drug administration to immigrant agricultural workers in the outbreak epicenter [21,22]. The mass drug administration program (2013-2014) involved administering chloroquine and primaquine under direct observation to all consenting immigrants in affected areas. Following comprehensive control implementation, locally acquired cases declined steadily, with none reported during 2022-2024.

The key milestones in Greece’s malaria eradication journey are given in Table 1.

**Table 1:** Key Milestones in Greece’s Malaria Eradication Journey DDT: dichlorodiphenyltrichloroethane

Year	Event	Significance
1905	Major epidemic; ~960,000 cases	Catalyzed an organized public health response
1905	Greek Anti-Malaria League established	First institutional framework for control
1908	Quinine monopoly (Law 3252)	Ensured affordable treatment access
1922-1923	Refugee crisis; 70% malaria mortality	Unprecedented public health emergency
1920s-1930s	Rockefeller Foundation initiatives	International technical assistance
1946	National eradication campaign launched	Comprehensive multi-intervention strategy
1950s	DDT indoor residual spraying begins	Cornerstone of vector control
1974	WHO certification: malaria-free	Official eradication achievement
1975	European region declared malaria-free	Historic regional milestone
2011-2012	*Plasmodium vivax* re-emergence (84 cases)	Demonstrated continued vulnerability
2013-2014	Mass drug administration response	Successful transmission interruption

Discussion

Greece’s successful elimination represents one of modern history’s most remarkable public health achievements, offering valuable lessons for contemporary efforts. Success resulted from multiple synergistic factors: scientific understanding enabling rational interventions, availability of practical tools (DDT, antimalarials, diagnostics), strong institutional capacity with trained personnel, sustained political commitment and financing across nearly three decades, and comprehensive integrated strategies [23].

The Greek experience demonstrates that integrated strategies are far more effective than single interventions. Simultaneous deployment of indoor residual spraying, mass treatment, environmental engineering, health education, and surveillance created synergistic effects that no single intervention could achieve. International cooperation accelerated progress, with the Rockefeller Foundation, League of Nations, and WHO providing financial resources and technical expertise.

For contemporary elimination efforts, Greece’s experience highlights key lessons. First, sustained commitment over decades is essential-elimination requires generational dedication across political cycles. Second, comprehensive approaches integrating multiple interventions are indispensable. Third, building local capacity through training provides sustainable foundations outlasting external assistance. Fourth, international cooperation remains vital, though ultimate success must rest with national programs. Finally, addressing social determinants alongside biomedical interventions is crucial for sustainable elimination [24].

The 2011-2012 outbreak demonstrates that vigilance must continue indefinitely. Elimination does not guarantee permanent security without continued surveillance and rapid response capacity. The outbreak revealed how quickly transmission can resume when imported infections encounter competent vectors, even after three decades without indigenous transmission. The persistence of indigenous *A. sacharovi *populations, despite decades without local transmission, underscores the biological challenge. These vectors maintained their competence to transmit *P. vivax*, requiring only the introduction of infected human hosts to re-establish transmission cycles. This biological reality, combined with increasing global migration from malaria-endemic regions, creates an ongoing reintroduction risk that demands sustained surveillance infrastructure.

Globalization and migration create new challenges; infections imported by mobile populations can re-establish transmission if vectors persist, demanding that malaria-free countries maintain specialized expertise and surveillance systems. Greece’s elimination occurred under favorable conditions not universally present in current endemic regions: relatively strong health infrastructure, substantial international assistance, political stability, and a temperate climate limiting seasonal transmission. Many endemic countries face more difficult circumstances: weak infrastructure, political instability, tropical climates permitting year-round transmission, and higher baseline intensity.

Additionally, biological challenges have intensified, widespread antimalarial drug resistance and insecticide resistance among vectors threaten programs relying on interventions that proved decisive in Greece. The widespread emergence of pyrethroid resistance in *Anopheles* populations globally, including in the Eastern Mediterranean region, where resistance has been documented in *A. stephensi*, poses particular concern [25,26]. Greece’s successful use of DDT cannot be simply replicated, given contemporary insecticide resistance patterns and environmental concerns. These challenges require innovative solutions, including new drugs, insecticides, and potentially vaccines unavailable during Greece’s campaign.

Looking forward, as the global community pursues WHO’s 2030 elimination targets, Greece’s experience provides inspiration and practical guidance while cautioning against excessive optimism. Elimination is achievable; Greece and many countries have proven this [27]. However, success depends on sustained effort, adequate resources, comprehensive strategies, strong institutions, and political commitment across decades. New tools offer opportunities unavailable during Greece’s campaign, yet will be effective only when deployed within resilient health systems supported by political will and stable financing. Technology alone cannot substitute for sustained commitment, institutional strength, and comprehensive strategies that characterized Greece’s successful campaign.

## Conclusions

The eradication of malaria from Greece stands as an enduring testament that ancient scourges can be conquered through science, determination, and collective action. From Hippocrates’ descriptions to the final WHO certification, Greece’s malaria story spans millennia. The successful elimination demonstrates that comprehensive, sustained public health interventions can overcome deeply entrenched endemic diseases. However, there are sobering reminders that elimination is not permanent without continued vigilance. Maintaining malaria-free status requires ongoing surveillance, rapid response capacity, and elimination of reservoirs, enabling transmission reestablishment.

For countries pursuing malaria elimination, Greece’s experience offers both hope and practical lessons. The challenge is formidable but achievable with sustained commitment, adequate resources, comprehensive approaches, and political will maintained across generations. As the global community works toward the WHO’s 2030 targets, Greece’s century-long effort provides valuable guidance for contemporary global health challenges. The ultimate lesson is one of possibility; ancient diseases can be eliminated through sustained scientific, political, and social action, offering hope that continued global efforts can eventually free humanity from malaria’s burden.
